# Supplementary material for: Adenoviral-Vectored Multivalent Vaccine Provides Durable Protection Against Influenza B Viruses from Victoria-like and Yamagata-like Lineages
Source: Int J Mol Sci. 2025 Feb 12;26(4):1538. doi: 10.3390/ijms26041538 (PMC11855595; doi:10.3390/ijms26041538)
Supplement: Supplementary file 1 [file ijms-26-01538-s001.zip › ijms-3457463-supplementary.pdf]

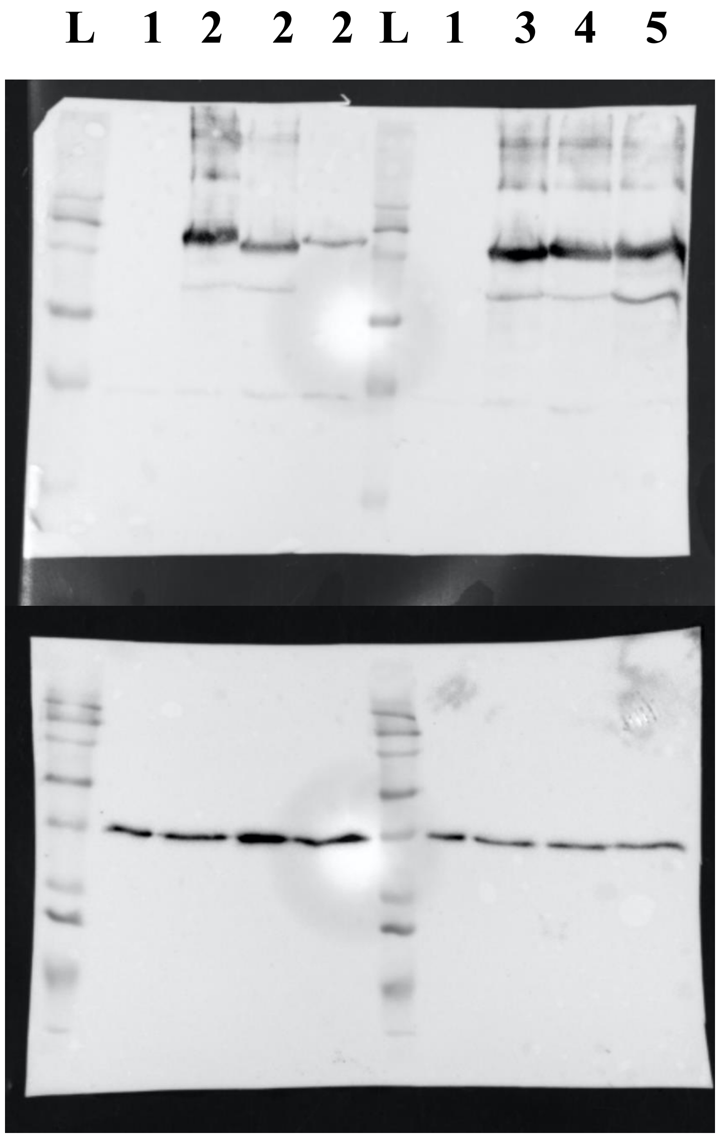

Figure S1. Full Western blot images from which Figure 1B were taken.
Lanes correspond to L) ladder, 1) uninfected control cells, 2) unrelated B/HA-infected cells 3) B/Vermont/2/2012 HA infected cells, 4) B/Malaysia/2506/2004 HA infected cells, or 5) B/Victoria/2/1987 HA infected cells. Anti-HA polyclonal serum was used as a primary antibody for top blot, anti-GAPDH monoclonal antibody was used for bottom blot.

Figure S2. IBV Protein Sequence Alignment. The differences in amino acid sequence of the proteins used in this study are shown in the ClustalW alignment. The conserved and variable regions are easily identified, and the amino acid positions are denoted on top of the alignment. rAd-Tri-Vic vaccine strains are denoted by a ^ and strains included in the 2018-2019 Fluzone® formulation used are denoted by a *.

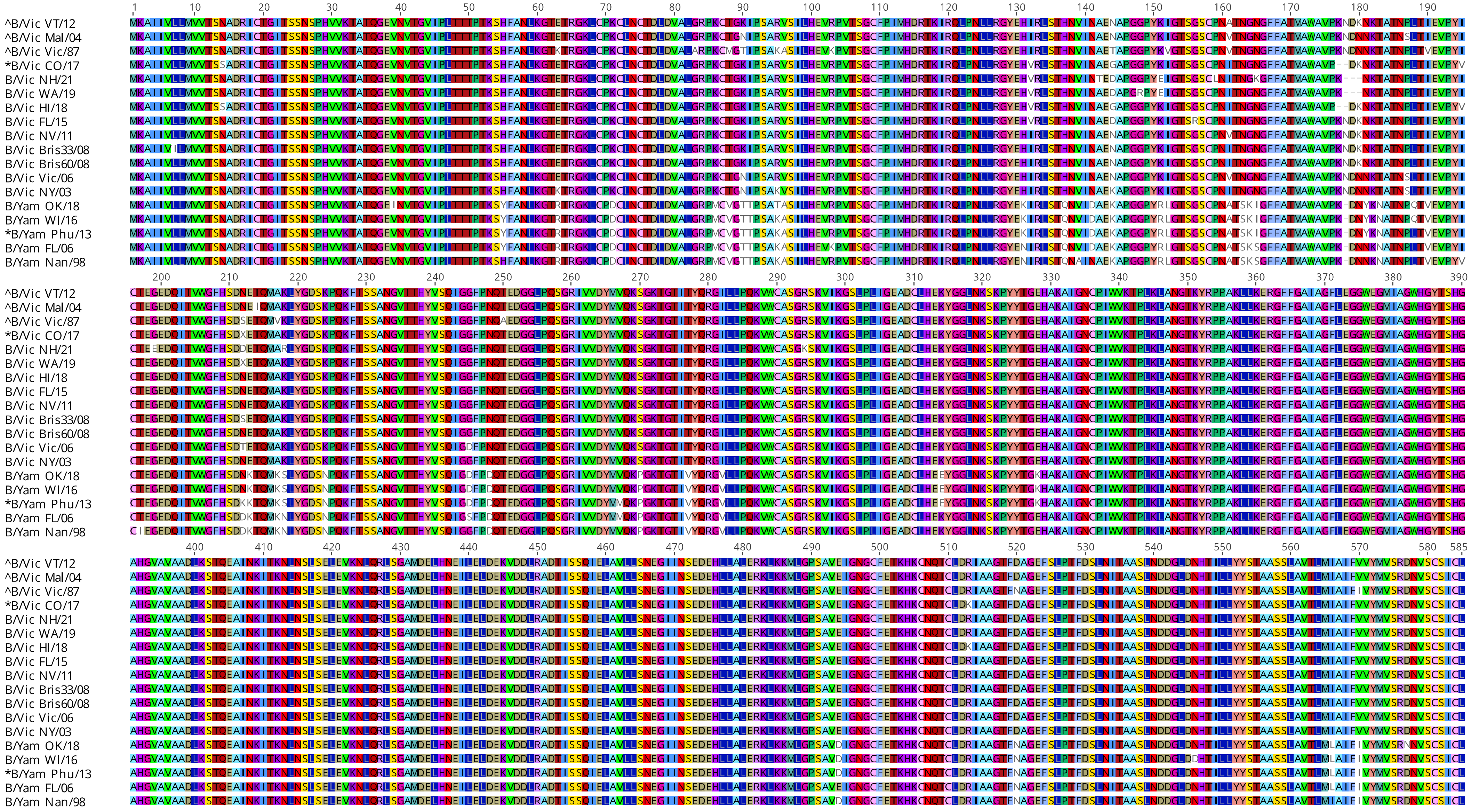

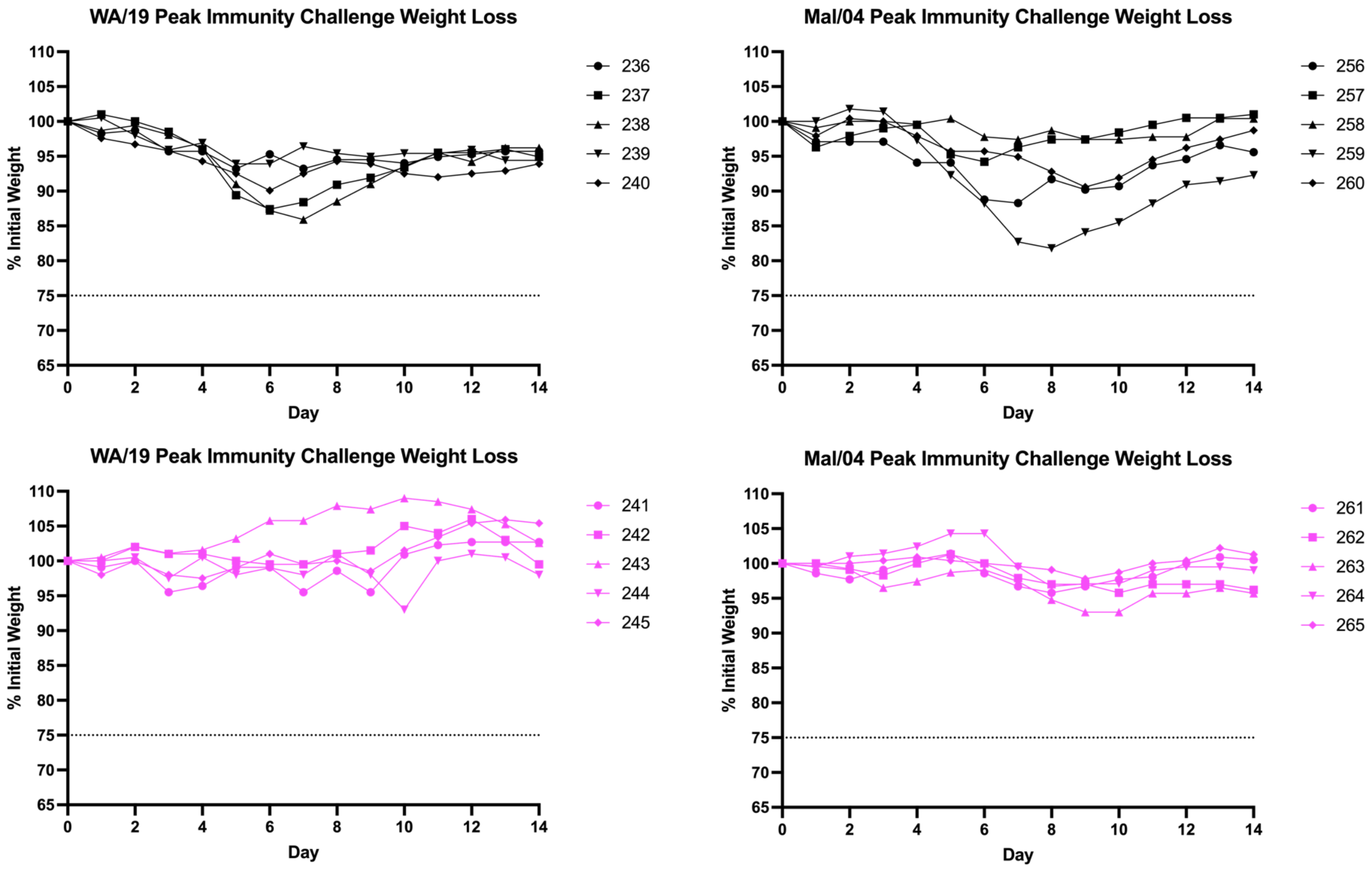

Figure S3. Individual mouse weights from B/Vic immediate immunity challenges. Individual mouse weights for Fluzone®-immunized mice (top) and B/Vic WT HA-immunized mice (bottom) used to calculate AUC values. The distance of each daily weight point from the 75% cutoff used for human euthanasia was used to calculate the area under the curve for each individual mouse, designated by the symbol and lines over the 14 days.

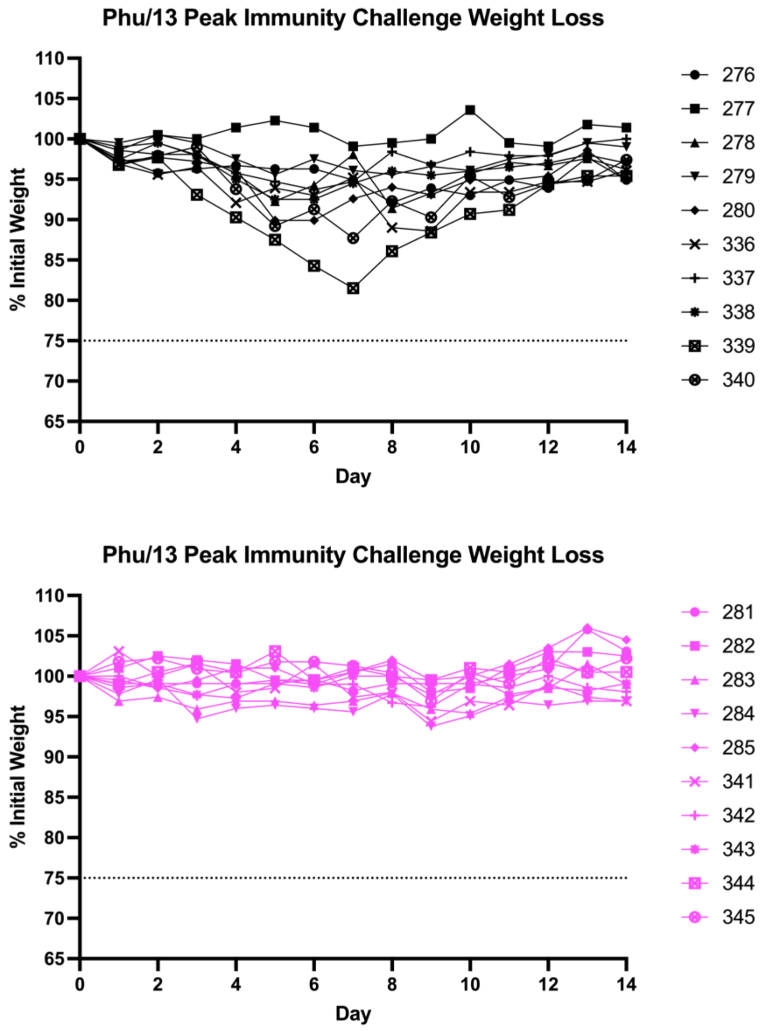

Figure S4. Individual mouse weights from B/Vic immediate immunity challenges. Individual mouse weights for Fluzone®-immunized mice (top) and B/Vic WT HA-immunized mice (bottom) used to calculate AUC values. The distance of each daily weight point from the 75% cutoff used for human euthanasia was used to calculate the area under the curve for each individual mouse, designated by the symbol and lines over the 14 days.

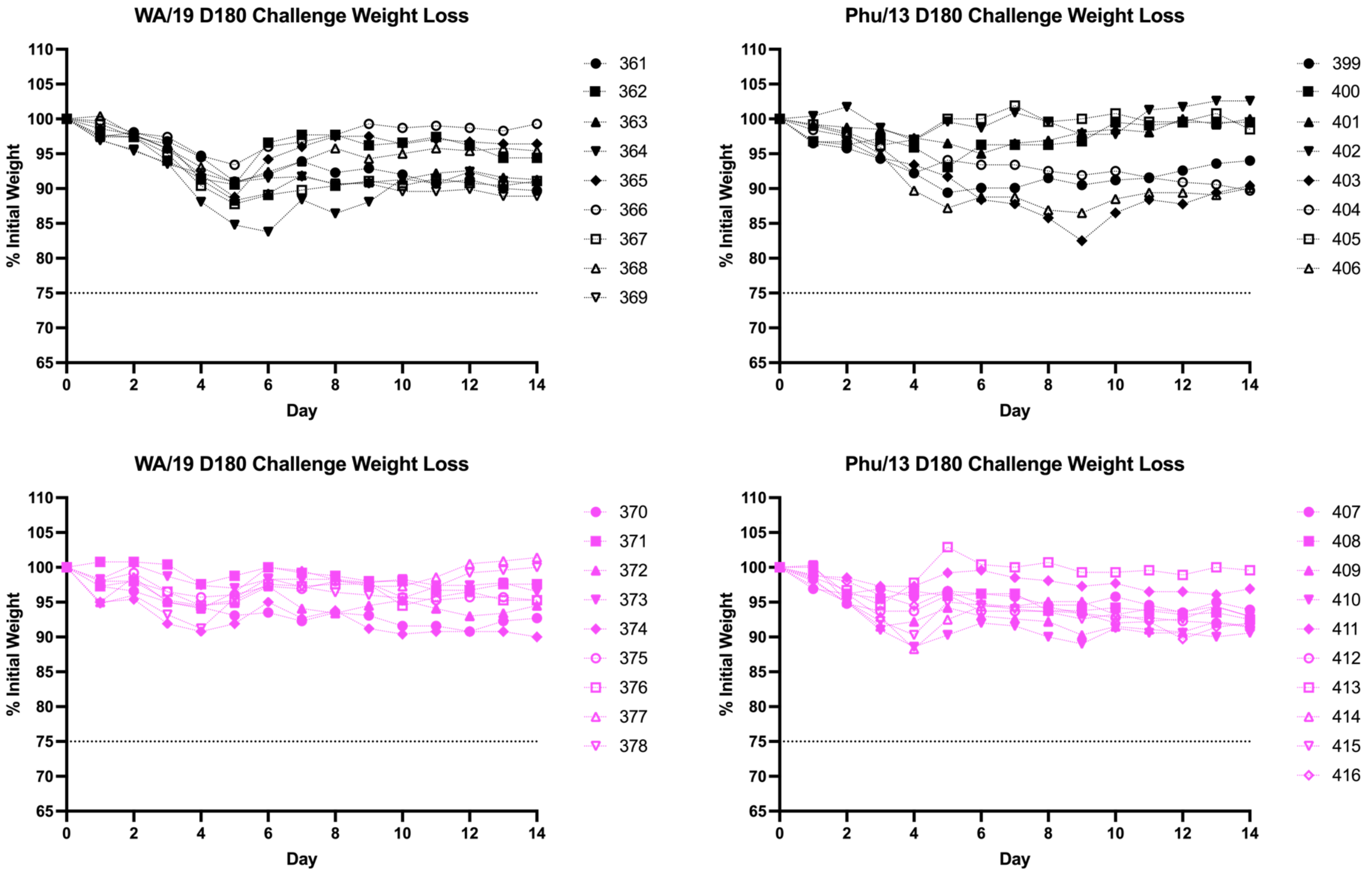

Figure S5. Individual mouse weights from B/Vic immediate immunity challenges. Individual mouse weights for Fluzone®-immunized mice (top) and B/Vic WT HA-immunized mice (bottom) used to calculate AUC values. The distance of each daily weight point from the 75% cutoff used for human euthanasia was used to calculate the area under the curve for each individual mouse, designated by the symbol and lines over the 14 days.
